# Splice‐switch oligonucleotide‐based combinatorial platform prioritizes synthetic lethal targets CHK1 and BRD4 against MYC‐driven hepatocellular carcinoma

**DOI:** 10.1002/btm2.10363

**Published:** 2022-09-03

**Authors:** Dexter Kai Hao Thng, Tan Boon Toh, Paolo Pigini, Lissa Hooi, Yock Young Dan, Pierce Kah‐Hoe Chow, Glenn Kunnath Bonney, Masturah Bte Mohd Abdul Rashid, Ernesto Guccione, Dave Keng Boon Wee, Edward Kai‐Hua Chow

**Affiliations:** ^1^ Cancer Science Institute of Singapore, National University of Singapore Singapore Singapore; ^2^ The N.1 Institute for Health, National University of Singapore Singapore Singapore; ^3^ The Institute for Digital Medicine (WisDM), Yong Loo Lin School of Medicine, National University of Singapore Singapore; ^4^ Institute of Molecular and Cell Biology (IMCB), Agency for Science, Technology and Research (A*STAR) Singapore Singapore; ^5^ NUS Centre for Cancer Research (N2CR), Yong Loo Lin School of Medicine National University of Singapore Singapore Singapore; ^6^ Division of Gastroenterology and Hepatology National University Health System Singapore Singapore; ^7^ Department of Medicine, Yong Loo Lin School of Medicine National University of Singapore Singapore Singapore; ^8^ Division of Surgical Oncology National Cancer Centre Singapore Singapore Singapore; ^9^ Department of Hepato‐Pancreato‐Biliary and Transplant Surgery Singapore General Hospital Singapore Singapore; ^10^ Duke‐NUS Medical School Singapore Singapore; ^11^ Division of Hepatobiliary and Liver Transplantation Surgery National University Health System Singapore Singapore; ^12^ KYAN Therapeutics Singapore Singapore; ^13^ Department of Oncological Sciences Tisch Cancer Institute, Icahn School of Medicine at Mount Sinai New York New York USA; ^14^ Mount Sinai Center for Therapeutics Discovery, Department of Oncological and Pharmacological Sciences Icahn School of Medicine at Mount Sinai New York New York USA; ^15^ Department of Pharmacology, Yong Loo Lin School of Medicine National University of Singapore Singapore Singapore; ^16^ Department of Biomedical Engineering, College of Design and Engineering National University of Singapore Singapore Singapore

**Keywords:** MYC synthetic lethality, quadratic phenotypic optimization platform, RNA therapeutics, splice‐switch oligonucleotides

## Abstract

Deregulation of MYC is among the most frequent oncogenic drivers in hepatocellular carcinoma (HCC). Unfortunately, the clinical success of MYC‐targeted therapies is limited. Synthetic lethality offers an alternative therapeutic strategy by leveraging on vulnerabilities in tumors with MYC deregulation. While several synthetic lethal targets of MYC have been identified in HCC, the need to prioritize targets with the greatest therapeutic potential has been unmet. Here, we demonstrate that by pairing splice‐switch oligonucleotide (SSO) technologies with our phenotypic‐analytical hybrid multidrug interrogation platform, quadratic phenotypic optimization platform (QPOP), we can disrupt the functional expression of these targets in specific combinatorial tests to rapidly determine target–target interactions and rank synthetic lethality targets. Our SSO‐QPOP analyses revealed that simultaneous attenuation of CHK1 and BRD4 function is an effective combination specific in MYC‐deregulated HCC, successfully suppressing HCC progression in vitro. Pharmacological inhibitors of CHK1 and BRD4 further demonstrated its translational value by exhibiting synergistic interactions in patient‐derived xenograft organoid models of HCC harboring high levels of MYC deregulation. Collectively, our work demonstrates the capacity of SSO‐QPOP as a target prioritization tool in the drug development pipeline, as well as the therapeutic potential of CHK1 and BRD4 in MYC‐driven HCC.

**List of abbreviations**
CI: combination index
C_max_
: maximum concentrations of drug available in the blood after administration
DRI: dose reduction index
GDC TCGA‐LIHC: Genomic Data Commons The Cancer Genome Atlas Liver Hepatocellular Carcinoma
HCC: hepatocellular carcinoma
IC_50_
: half‐maximal inhibitory concentration
OACD: orthogonal array composite design
PDX: patient‐derived xenograft
PDXO: patient‐derived xenograft organoids
QPOP: quadratic phenotypic optimization platform
SSO: splice‐switch oligonucleotide

## 1 INTRODUCTION

MYC is a general transcription factor that functions as a master regulator of cell cycle. As an oncogene, MYC is frequently deregulated across various cancer types, including breast cancer, liver cancer, colorectal carcinoma, multiple myeloma, and lymphomas, frequently inducing a dependency on the oncogene for disease progression.^1^, ^2^, ^3^, ^4^, ^5^, ^6^ These tumors are collectively referred to as MYC‐driven/deregulated tumors. However, directly targeting MYC has proven to be a challenge in the clinics owing to its general function as a transcription factor necessary for normal cellular physiology, and the absence of a defined three‐dimensional pocket for the design of small‐molecule inhibitors.^7^, ^8^ There is therefore an unmet need in developing cancer therapies against MYC. To overcome the undruggable nature of MYC, synthetic lethality offers an ideal treatment strategy, where vulnerabilities in MYC‐deregulated cancers are leveraged as therapeutic targets.^9^ Two key groups of MYC synthetic lethal targets are regulators of *MYC* transcription (e.g., BRD4 and CDK9) and MYC stability (e.g., aurora kinases A and B, and polo‐like kinase 1), regulating the levels of MYC in tumor cells.^3^, ^10^, ^11^, ^12^, ^13^ MYC vulnerabilities have also been identified in other pathways such as cell proliferation (e.g., CDK1), evasion of apoptosis (e.g., MCL1 and CHK1), cellular metabolism (e.g., glutaminase‐1 and LDHA), and biosynthesis (e.g., IMPDH2 and ribosomal DNA transcription).^14^, ^15^, ^16^, ^17^, ^18^, ^19^, ^20^ Attempts at impeding MYC function have also been directed at targeting MAX, the main co‐transcription factor necessary for MYC‐dependent transcription.^21^, ^22^, ^23^ Despite the plethora of MYC synthetic lethal targets and development of small‐molecule inhibitors against them, targeted synthetic lethality therapy against MYC‐driven cancers is still not approved for clinical use.^9^ Furthermore, it is unlikely that inhibition of each synthetic lethality target is equal in different cancer types where MYC drives pathogenesis. Hence, there is a need to prioritize the synthetic lethality vulnerabilities which offer the greatest therapeutic outcome against specific MYC‐driven cancers.

Notably, combination therapies have been gaining traction in cancer therapeutics against MYC due to inevitable acquired and polyclonal resistance against monotherapies.^9^ Tumors often acquire drug resistance through loss of drug‐to‐target binding site, activating alternative oncogenic pathways and expressing efflux pumps to remove the therapeutics. In addition, monotherapies cannot address the differential sensitivity of multiple clones to anticancer drugs due to the inherent heterogeneity within and between tumors.^24^ Thus, identifying pairs of synergistic vulnerabilities which are effective against MYC‐driven cancers may result in prolonging therapeutic durability and increasing the response rate.

Computational approaches have facilitated the discovery of novel therapeutic targets against cancers in the era of big data.^9^, ^25^, ^26^, ^27^ Leveraging on such computational platforms can therefore facilitate the process of target prioritization and early drug discovery. Here, we employ the use of the quadratic phenotypic optimization platform (QPOP) to characterize and narrow down effective combinations of targets which mediate the best possible treatment outcomes in MYC‐driven hepatocellular carcinoma (HCC) as our disease model of choice. QPOP is an experimental–analytical hybrid platform which utilizes the phenotypic response of biological systems to a set of predefined combination of drugs and dosages as the input data for the establishment of a second‐order regression model and corresponding parabolic surfaces.^28^ QPOP and other similar approaches are premised on the discovery that responses of biological systems to external perturbations can be modeled accurately and robustly with a second‐order polynomial equation, and that the effects of any higher‐order components are negligible.^28^, ^29^, ^30^, ^31^, ^32^

As a phenotypic‐driven and unbiased platform, QPOP streamlines the drug combination identification pipeline without any reference to the mechanisms of action of the chosen drugs nor predetermined drug synergism. An orthogonal array composite design (OACD) is utilized to curate the combinations of drugs and dosages against which the cancer cells are screened. To identify the optimal drug combination within a set of six drugs over a range of three concentrations, samples are screened with 50 combinations using the OACD instead of all 3^6^ (729) possible combinations, significantly streamlining the drug combination identification pipeline. The OACD is introduced by merging the two‐level fractional factorial design and three‐level orthogonal array in a single composite study as described previously.^33^ This greatly improves the capacity for factor screening and in‐depth analyses. In addition, OACD offers a balanced trade‐off between estimation efficiency of the model and run‐size economy, offering a viable alternative to other composite designs such as central composite design.^33^ Phenotypic responses of samples to the OACD combinations are subsequently used to establish a quadratic regression model from which the phenotypic response of biological system to all possible combination of drugs and doses are determined. Prior studies have also demonstrated that a quadratic regression model outperforms neural networks in modeling the nonlinear nature of cellular responses.^29^, ^34^ Given a panel of drugs and dosages, QPOP is therefore able to rapidly identify the globally optimized drug combination parameters which yields the greatest therapeutic outcome of interest. Here, we hypothesize that by using dose‐dependent RNA therapeutics such as antisense and splice‐switching oligonucleotides (ASOs/SSOs) which disrupt target expression at the transcriptional level in place of small molecule inhibitors, QPOP has the additional capacity to prioritize gene targets with the greatest therapeutic capabilities and guide early drug discovery.

ASOs are emerging RNA therapeutics, which exhibit anticancer properties by binding to their specific oncogene targets and manipulating its expression levels. ASO‐based therapies function through two different mechanisms—by inducing RNAse‐H‐dependent mRNA cleavage and by modulating of pre‐mRNA splicing events to regulate gene expression.^35^ ASOs which act through the latter mechanism are specifically termed as SSOs. SSOs are chemically modified single‐stranded RNAs which bind specifically to the splice sites of target mRNAs, to effect steric hindrance and impede splicing events.^36^, ^37^ Consequently, SSO‐induced exon skipping results in functional knockdown of the target gene and possibly nonsense‐mediated decay of the target.^38^ To date, 10 oligonucleotide‐based drugs have received approval by the FDA for clinical use, of which half are SSOs.^39^, ^40^, ^41^, ^42^, ^43^, ^44^ Recent advances in SSO technologies have also demonstrated the value of SSOs as candidate RNA therapeutics with significant anticancer properties in *in vivo* models of HCC, melanoma, prostate, and lung cancer.^38^, ^45^, ^46^, ^47^ Additionally, prior applications of SSOs have exhibited dose‐dependent splice‐switch events and is therefore suitable for use in conjunction with QPOP for target prioritization, where the dosages of the therapeutics are considered.^48^

Here, we report the development and the use of SSO‐QPOP that exhibits a novel function in the target prioritization stage of the drug development pipeline by leveraging on genetic modulators. We propose that the SSO‐QPOP‐derived combination of CHK1 and BRD4 is a pair of MYC synthetic lethal targets with the greatest therapeutic potential in the treatment of MYC‐driven HCC. Concurrent inhibition of both targets via SSO therapeutic or pharmacological agents offers a novel and effective therapeutic strategy against MYC‐driven cancer, particularly MYC‐driven liver cancer.

## 2 RESULTS

### 2.1 Quadratic phenotypic drug and splice‐switch oligonucleotides combination optimization against MYC‐deregulated HCC

**FIGURE 1:**
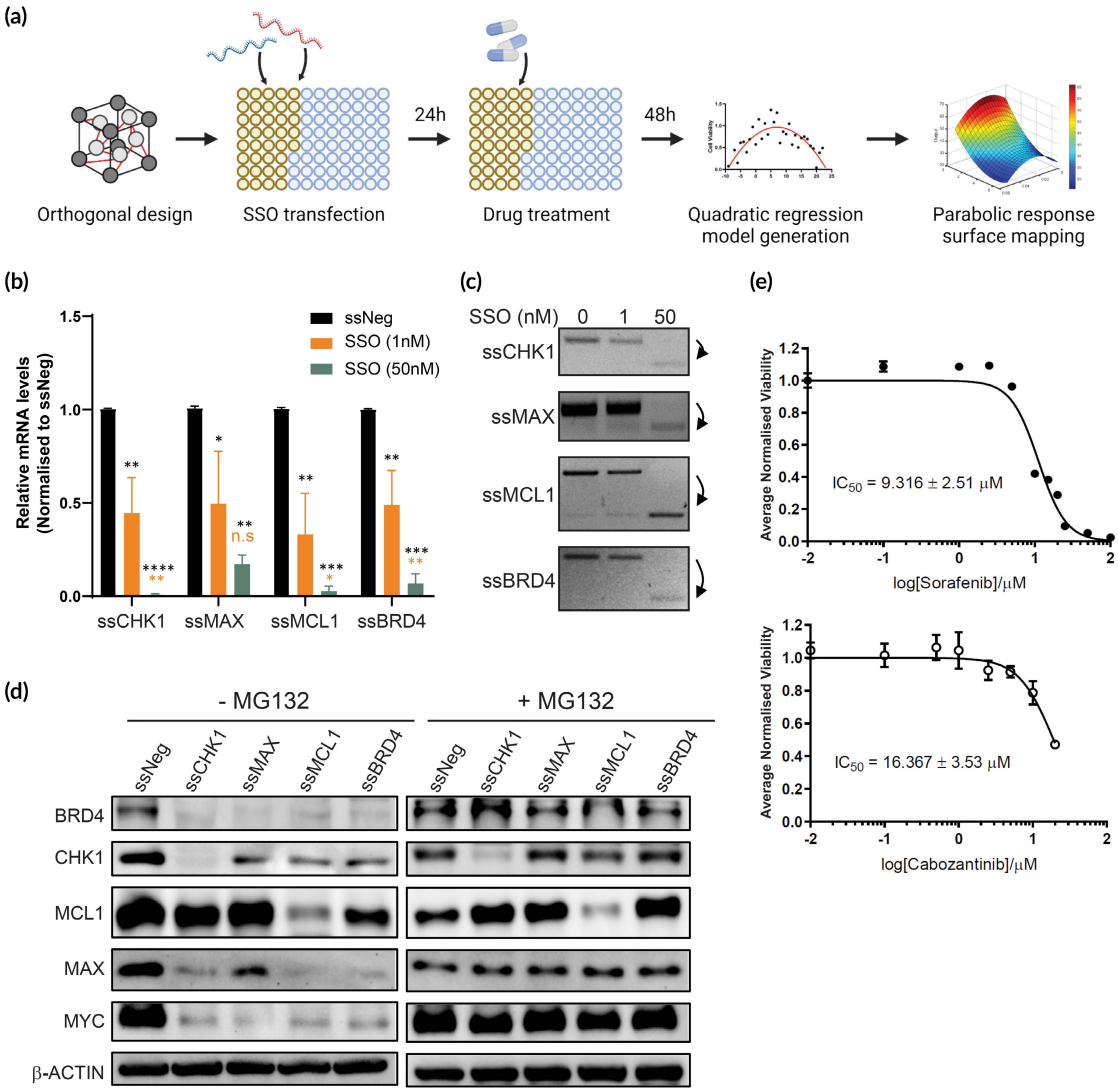
Establishment of splice‐switch oligonucleotide (SSO)‐based quadratic phenotypic optimization platform (QPOP) combination optimization pipeline in Bel7402 cell line. (a) Workflow of SSO combination optimization via QPOP analysis. Experimental combinations of SSO and standard‐of‐care drugs were predetermined using orthogonal array composite design. Cells were co‐transfected with SSO combinations and then drug combinations 24 h after. Cell viabilities were determined 48 h after and used to generate a second‐order regression model for parabolic response surface mapping via QPOP analysis. Figure was created with BioRender.com. (b) Validation of splicing events in SSO target genes. Relative expressions of spliced transcripts were determined via quantitative real time‐polymerase chain reaction and (c) gel electrophoresis following SSO transfection at 1 and 50 nM. Ordinary one‐way ANOVA and Tukey's pairwise comparisons were performed as recommended (n.s.: not significant; *p < 0.05; **p < 0.01; ***p < 0.001). (d) Functional consequence of SSO transfection on protein expression levels of target genes and MYC were determined by immunoblotting. Proteasome inhibitor treatment with MG132 for 6 h posttransfection was performed to determine the specific effect of SSO on transcriptional and translational regulation of target gene expression. (e) Dose–response curves of standard‐of‐care drugs sorafenib and cabozantinib as quantified via MTS. IC_50_ values are represented as means ± SD (*n* = 3).

We sought to identify synergistic gene pairs amongst a panel of MYC synthetic lethal targets via SSO‐QPOP experiments. A schematic of the overall experimental design is summarized in Figure 1a. A six‐modulator three‐level SSO‐QPOP analysis comprising of 50 combinations derived from an orthogonal array composite design (OACD) was performed (Table S1).^33^ The panel of modulators include the standard‐of‐care therapies for HCC, sorafenib and cabozantinib, as well as SSOs against four synthetic lethal genes of MYC–*CHEK1*, *MAX*, *MCL1*, and *BRD4* (ssCHK1, ssMAX, ssMCL1, ssBRD4).^10^, ^15^, ^16^, ^23^, ^49^, ^50^

SSOs were designed and generated to alter the functional domains of the target proteins at specific sites (Figure S1A). Successful splice‐switch by the SSOs were determined in our cell line model, Bel7402, via quantitative real‐time polymerase chain reaction. Relative expression of the spliced transcripts demonstrated that the four SSOs induced a dose‐dependent splicing event, with an average splicing rate of 55% and 95% at 1 nM and 50 nM of SSO, respectively (Figure 1b,c). The four SSOs were selected from a panel of eight SSOs, each targeting a different vulnerability previously identified in MYC‐driven cancers, due to their higher splicing efficiencies (Figure S1B). Expression levels of the total transcripts indicated that ssCHK1 and ssMCL1 transfection induced a significant reduction in the total gene transcript levels, further enhancing the functional knockdown of the target genes (Figure S1C). A significant downregulation of MYC protein levels was induced by all four SSOs, and crosstalk between the targets were evident from the downregulation of CHK1, BRD4 and MAX across almost all four SSO treatment (Figure 1d). SSO specificity was then determined by adding proteasome inhibitor MG132 posttransfection. Similar to the total transcript level, only ssCHK1 and ssMCL1 saw a significant downregulation in its target protein expression, with no crosstalk between the SSOs (Figures 1d and S1D).

To determine the appropriate dosages of the two small‐molecule inhibitors, sorafenib and cabozantinib, we first performed a single drug dose–response assay to determine the half‐maximal inhibitory concentrations (IC_50_) of each drug (Figure 1e). IC_15_ and IC_30_ concentrations were used in the SSO‐QPOP combination treatments if the IC_50_ was lower than the highest concentrations of each drug available in the blood when administered in patients (C_max_).^51^ Alternatively, the 5% and 10% C_max_ will be used in the SSO‐QPOP combination treatments to ensure that the drug dosages are aligned with the clinically relevant concentrations (Table S2). Lastly, optimization of the SSO‐QPOP conditions demonstrated that the transcripts were sufficiently spliced 24 h posttransfection, achieving at least a 75% splicing efficiency across all four SSOs without significantly altering the viability of the cells (Figure S1E). SSO‐QPOP combination treatment was therefore performed by first transfecting the cells with the SSO in combination prior to treatment with sorafenib/cabozantinib combinations 24 h after (Figure 1a).

### 2.2 SSO‐QPOP identified CHK1 and BRD4 combinations as promising synthetic lethal targets of MYC

**FIGURE 2:**
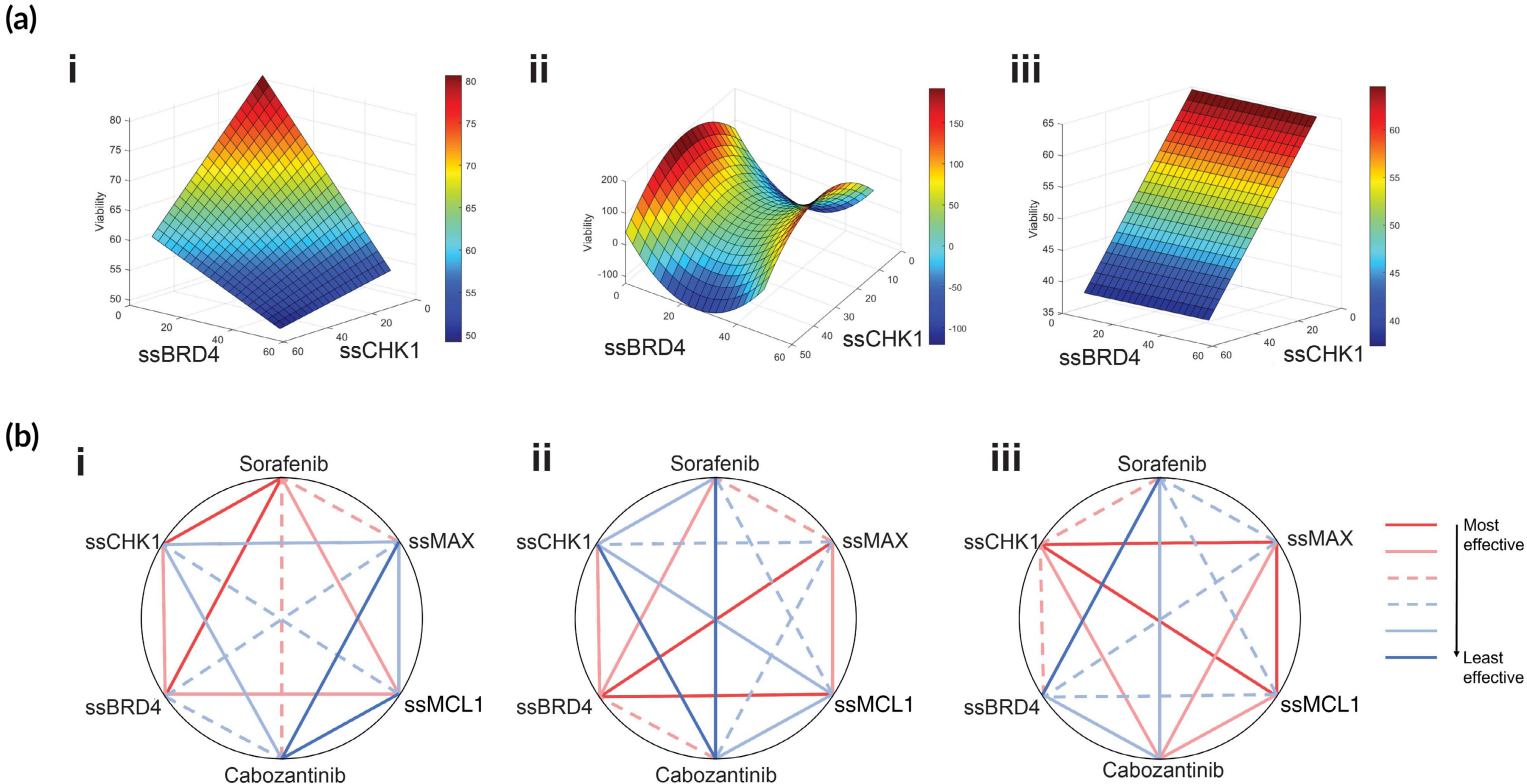
Quadratic phenotypic optimization platform (QPOP)‐identified combination ssCHK1 and ssBRD4 is an effective combination against MYC‐deregulated hepatocellular carcinoma (HCC). (a) Response surface mapping of interactions between ssCHK1 and ssBRD4 in (i) MYC^Hi^ Bel7402, (ii) HCCLM3, (iii) and MYC^Lo^ SNU387. (b) Polygonograms illustrating the efficacy interactions of all two‐drug/SSO combinations in (i) MYC^Hi^ Bel7402, (ii) HCCLM3, (iii) and MYC^Lo^ SNU387. Efficacies of each combination are represented as geometric means and ranked based on the percentiles of each combination. (red, most effective, 83.3–100th percentile; pink, second most effective, 66.7–83.3th percentile; pink and dotted, third most effective, 50–66.7th percentile; light blue and dotted, third least effective, 33.3‐50th percentile; light blue, second least effective, 16.7–33.3th percentile; dark blue, least effective, 0–33.3th percentile)

Utilizing the OACD, three HCC cell lines, Bel7402, HCCLM3, and SNU387, were then treated with the 50 unique SSO‐QPOP combinations for the analysis (Table S1).^33^ Bel7402 and HCCLM3 are representative models of HCC with MYC deregulation, harboring elevated levels of MYC (MYC^Hi^), while SNU387 is representative of HCC with low levels of MYC expression (MYC^Lo^) (Figure S2A). The viability of the cells 48 h posttreatment was used as input variables for the SSO‐QPOP response surface mapping. The second‐order regression model for all three lines exhibited a good fit, with *R* ^2^ values above 0.8 (Table S3). Notably, the combination of ssCHK1 and ssBRD4 was among the top‐ranked two‐modulator combinations in both MYC^Hi^ lines, Bel7402 and HCCLM3, outperforming the standard‐of‐care drugs, while being absent in top‐ranked combinations for MYC^Lo^ SNU387 (Table 1). The bilinear effect of ssCHK1 and ssBRD4 was also significant in only the MYC^Hi^ lines (Table S3). Interrogation into the parabolic response surface maps for the combination of ssCHK1 and ssBRD4 derived from the second‐order regression model reflects a synergistic interaction in MYC^Hi^ lines Bel7402 and HCCLM3. This is illustrated by a steep decrease in the predicted viability of the cells with increasing concentrations of both SSOs (Figure 2ai,ii). Conversely, MYC^Lo^ SNU387 was expected to respond only to increasing concentrations of ssCHK1 and not ssBRD4 (Figure 2aiii).

**TABLE 1:** Top 10 two‐modulator ranked combinations for Bel7402, HCCLM3, and SNU387

Top‐ranked 2‐SSO combinations in MYC^Hi^ Bel7402								
Rank	Sorafenib	Cabozantinib	ssCHK1	ssMAX	ssMCL1	ssBRD4	Viability	
1 (112)	0	0	0	0	50	50	0.296	
2 (151)	6.461	0	0	0	50	0	0.303	
3 (187)	0	0	50	0	0	50	0.331	ssCHK1 + ssBRD4
4 (196)	6.461	0	0	0	0	50	0.339	
5 (226)	0	0	50	0	50	0	0.361	
6 (256)	4.417	0	0	0	50	0	0.381	
7 (271)	4.417	0	0	0	0	50	0.384	
8 (280)	6.461	0	50	0	0	0	0.386	
9 (301)	0	0	0	50	0	50	0.425	
10 (325)	4.417	0	50	0	0	0	0.45	
(484)	6.461	0	0	0	0	0	0.549	
(692)	0	0.2305	0	0	0	0	0.91	

Top‐ranked 2‐SSO combinations in MYC^Hi^ HCCLM3								
Rank	Sorafenib	Cabozantinib	ssCHK1	ssMAX	ssMCL1	ssBRD4	Viability	
1 (247)	0	0	0	50	50	0	0.261	
2 (282)	0	0	0	50	0	50	0.286	
3 (284)	0	0	50	50	0	0	0.286	
4 (306)	0	0	50	0	0	50	0.298	ssCHK1 + ssBRD4
5 (311)	0	0	0	0	50	50	0.302	
6 (315)	0	0	50	0	50	0	0.306	
7 (328)	0	0	0	50	0	1	0.313	
8 (342)	6.461	0	0	50	0	0	0.323	
9 (354)	0	0.461	0	50	0	0	0.334	
10 (373)	0	0	0	0	50	1	0.346	
(643)	6.461	0	0	0	0	0	0.697	
(657)	0	0.461	0	0	0	0	0.708	

Top‐ranked 2‐SSO combinations in MYC^Lo^ SNU387								
Rank	Sorafenib	Cabozantinib	ssCHK1	ssMAX	ssMCL1	ssBRD4	Viability	
1 (118)	0	0	50	50	0	0	0.292	
2 (136)	0	0	50	0	50	0	0.294	
3 (154)	0	0	0	50	50	0	0.312	
4 (217)	0	0.461	50	0	0	0	0.351	
5 (253)	0	0.461	0	0	50	0	0.356	
6 (316)	0	0.2305	50	0	0	0	0.377	
7 (325)	0	0.461	0	50	0	0	0.377	
8 (361)	0	0.2305	0	0	50	0	0.382	
9 (397)	0	0	50	1	0	0	0.401	
10 (406)	0	0	50	0	1	0	0.401	
(577)	0	0.461	0	0	0	0	0.629	
(723)	6.461	0	0	0	0	0	0.682	

*Note*: Overall rankings are in parentheses.

The differential sensitivities of the three cell lines to the panel of drugs and SSOs were further analyzed based on the QPOP‐projected viability outputs. The QPOP‐generated output values for all two‐drug/SSO combinations demonstrate that the ssCHK1‐ssBRD4 combination was more effective in MYC^Hi^ Bel7402 and HCCLM3, ranking among the top 25th percentile, but was only in the top 50th percentile for MYC^Lo^ SNU387 (Figure 2b). To evaluate the sensitivity of Bel7402 and HCCLM3 to ssCHK1 and ssBRD4 in comparison to SNU387, we further normalized the QPOP‐generated viability outputs of Bel7402 and HCCLM3 to SNU387 using the formula: Normalized viability = Viability_SNU387_ – Viability_Bel7402/HCCLM3_. The combination comprising both ssCHK1 and ssBRD4 remained amongst the top‐ranked combinations for both cell lines (Table S4). The collective SSO‐QPOP analyses indicate that MYC^Hi^ HCC exhibit greater sensitivity to the simultaneous targeting of CHK1 and BRD4.

### 2.3 Splice‐switch oligonucleotides targeting CHK1 and BRD4 induce MYC degradation and apoptosis in MYC^Hi^ HCC

**FIGURE 3:**
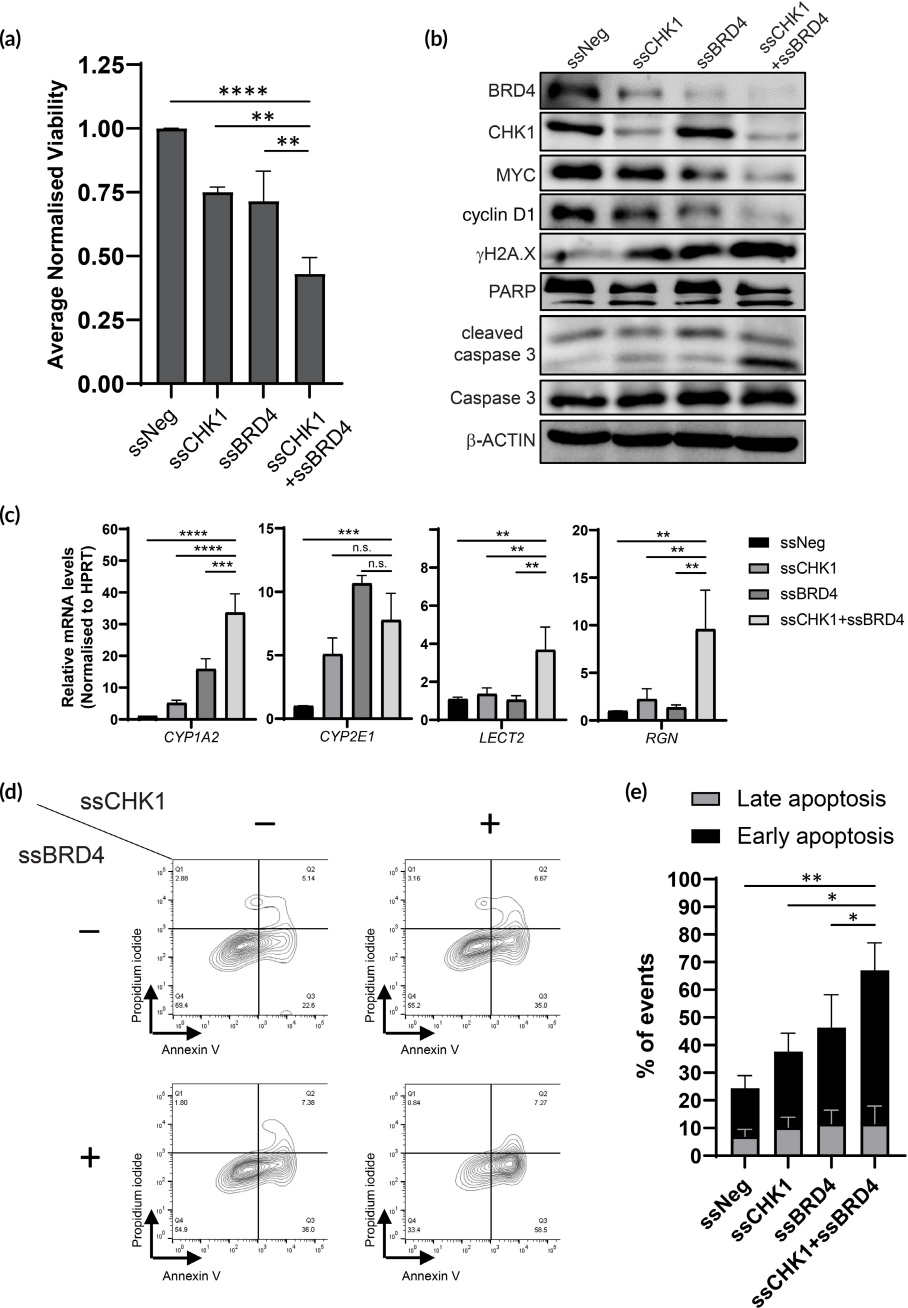
ssCHK1 and ssBRD4 co‐transfection exhibit antitumor properties in MYC^Hi^ Bel7402. (a) Validation of ssCHK1 and ssBRD4 in Bel7402 48 h after transfection by MTS. Ordinary one‐way ANOVA and Dunnett's pairwise comparison were performed as recommended (n.s.: not significant; *p < 0.05; **p < 0.01; ***p < 0.001). (b) Immunoblotting to determine the functional consequence on MYC activity, DNA damage, and apoptosis markers after ssCHK1 and ssBRD4 co‐transfection in Bel7402. (c) Relative gene expression of tumor suppressor genes in Bel7402 following single and combination therapy of ssCHK1 and ssBRD4 in Bel7402. Ordinary one‐way ANOVA and Dunnett's pairwise comparisons were performed as recommended (n.s.: not significant; *p < 0.05; **p < 0.01; ***p < 0.001). (d) Flow cytometric analysis of Annexin V apoptosis assay in Bel7402 co‐transfected with ssCHK1 and ssBRD4. (e) Quantification of apoptosis events from flow cytometric analysis in (d). One‐way ANOVA and Dunnett's pairwise comparisons were performed as recommended (n.s.: not significant; *p < 0.05; **p < 0.01; ***p < 0.001).

We proceeded to investigate the effects of the combination in Bel7402 to determine the efficacy of ssCHK1 and ssBRD4 in vitro. Concurrent treatment with both ssCHK1 and ssBRD4 induced a significant decrease in cell viability as compared to singly transfecting the cells (Figure 3a). On the contrary, poorly ranked combinations for Bel7402, such as ssMAX in combination with either ssMCL1 or ssCHK1 (overall rank 703/729 and 664/729 respectively), did not significantly reduce cell viability when used in combination (Figure S2B‐C). The viability of Bel7402 in response to these SSOs validated the efficacy of ssCHK1 and ssBRD4 in combination against HCC cells, as well as the robustness of the SSO‐QPOP analyses.

To evaluate the antitumor effects of targeting both CHK1 and BRD4, we performed immunoblotting on Bel7402 cells co‐transfected with ssCHK1 and ssBRD4 (Figure 3b). Notably, we observed that co‐transfection with ssCHK1 and ssBRD4 saw a greater reduction in MYC levels as compared to when the SSOs were administered singly. There was a corresponding decrease in the expression levels of cyclin D1, a key oncoprotein which functions in concert with MYC to drive cell cycle progression.^52^ qPCR analyses revealed a significant increase in the expression levels of several tumor suppressor genes in HCC, contributing to the anti‐tumor properties of ssCHK1 and ssBRD4 in impeding cell growth in vitro (Figure 3c). As CHK1 is a key component of the DNA damage repair pathway, and BRD4 has previously been shown to exhibit a novel function in facilitating replication stress‐induced DNA damage by oncogenes such as MYC, levels of DNA damage were determined in co‐transfected cells.^53^, ^54^, ^55^ Concurrent ablation of CHK1 and BRD4 resulted in a significant increase in DNA damage marker, γH2AX, as well as a corresponding increase in apoptosis markers, cleaved caspase 3, and cleaved PARP (Figure 3b). Flow cytometry analyses demonstrated a greater increase in the Annexin V^+^/propidium iodide^−^ cell population when transfected with both ssCHK1 and ssBRD4, synonymous with an increase in early apoptosis in vitro (Figure 3d,e). However, levels of Annexin V^+^/propidium iodide^+^ cells did not differ significantly. The levels of the various markers collectively indicate that the simultaneous inhibition of CHK1 and BRD4 via SSOs impeded MYC‐dependent cell growth, while initiating DNA damage‐induced apoptosis in vitro.

### 2.4 *MYC* , *CHEK1*, and *BRD4* expression portend poorer prognosis in HCC

**FIGURE 4:**
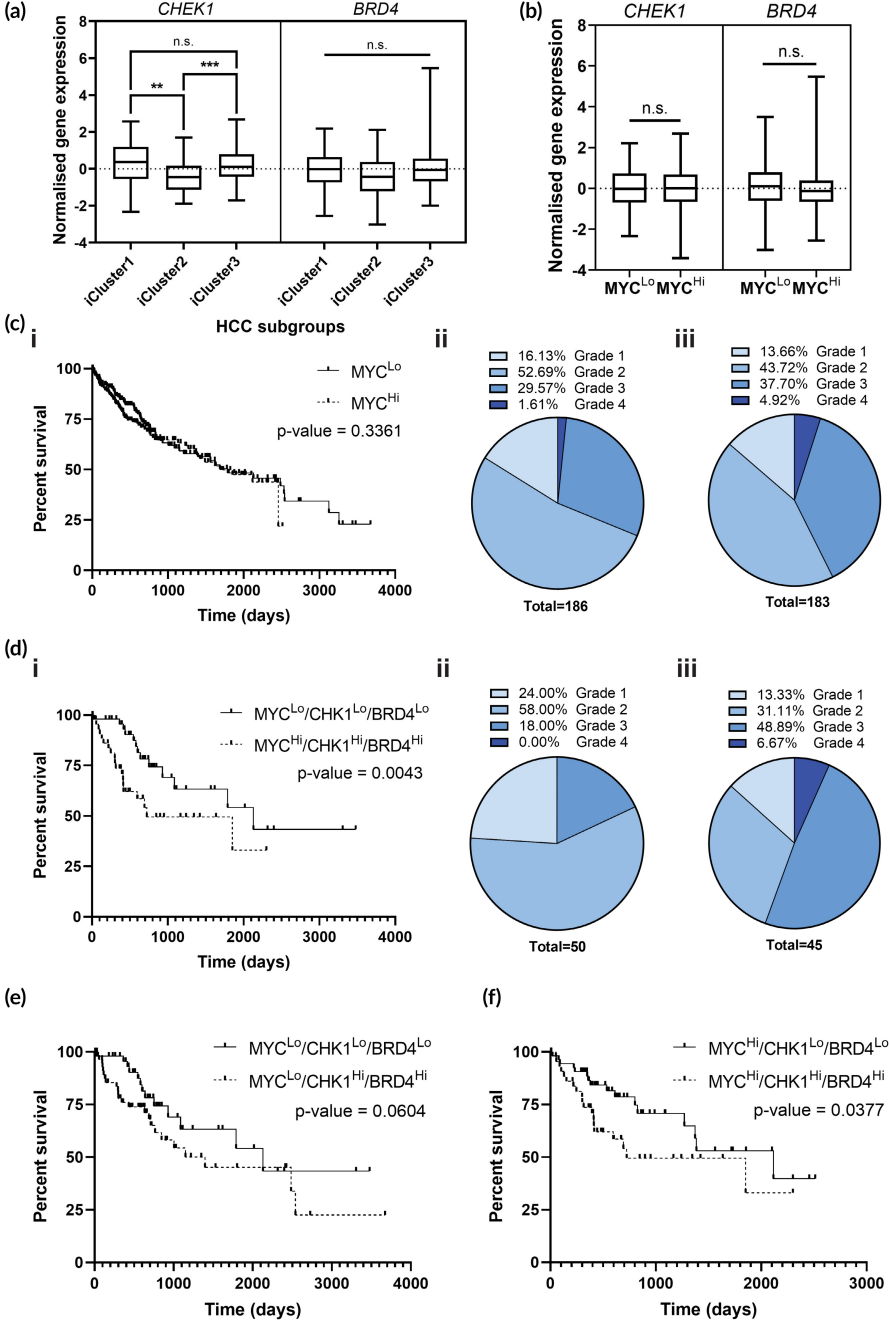
*CHEK1* and *BRD4* expression portends poorer prognosis in *MYC*‐stratified HCC. (a) Expression levels of *CHEK1* and *BRD4* in the Genomic Data Commons (GDC) The Cancer Genome Atlas Liver Hepatocellular Carcinoma (TCGA‐LIHC) patient cohort when stratified into three molecular subtypes (iCluster1‐3),^56^ (b) and when stratified into MYC^Lo^ and MYC^Hi^ tumors. Statistical analyses between molecular subtypes of HCC were performed using Games‐Howell corrected Brown–Forsythe and Welch ANOVA, and Dunn's corrected Kruskal–Wallis test for *CHEK1* and *BRD4* expression levels, respectively. Statistical analyses between MYC^Lo^ and MYC^Hi^ samples were performed using one‐sample *t*‐test. (n.s.: not significant; **p < 0.01; ***p < 0.001). (c) (i) Kaplan–Meier survival curves of overall patient survival according to expression levels of *MYC* from the GDC TCGA‐LIHC data set. (MYC^Lo^, *n* = 185; MYC^Hi^, *n* = 183). Distribution of (ii) MYC^Lo^ and (iii) MYC^Hi^ samples according to their neoplasm histologic grade. (d) (i) Kaplan–Meier survival curves of overall patient survival according to expression levels of *MYC*, *CHEK1* and *BRD4*. (MYC^Lo^/CHK1^Lo^/BRD4^Lo^, *n* = 50; MYC^Hi^/CHK1^Hi^/BRD4^Hi^, *n* = 44). Distribution of (ii) MYC^Lo^/CHK1^Lo^/BRD4^Lo^ and (iii) MYC^Hi^/CHK1^Hi^/BRD4^Hi^ samples according to their neoplasm histologic grade. (e) Kaplan–Meier survival curves of overall patient survival of MYC^Lo^ samples according to expression levels of *CHEK1* and *BRD4*. (MYC^Lo^/CHK1^Lo^/BRD4^Lo^, *n* = 50; MYC^Lo^/CHK1^Hi^/BRD4^Hi^, *n* = 59). (f) Kaplan–Meier survival curves of overall patient survival of MYC^Hi^ samples according to expression levels of *CHEK1* and *BRD4*. (MYC^Hi^/CHK1^Lo^/BRD4^Lo^, *n* = 55; MYC^Hi^/CHK1^Hi^/BRD4^Hi^, *n* = 44). Survival analyses were performed using Gehan–Breslow–Wilcoxon test.

To determine the clinical value of targeting CHK1 and BRD4 in MYC‐driven HCC, we assessed the role of *CHEK1* and *BRD4* expression in the pathogenesis of HCC in patients from the Genomic Data Commons (GDC) The Cancer Genome Atlas Liver Hepatocellular Carcinoma (TCGA‐LIHC) data set. Given the heterogeneous nature of HCC, we first evaluated the expression of both *CHEK1* and *BRD4* when HCC patient samples were stratified into three molecular subtypes, iCluster1‐3, by integrating multiomics analyses as described previously.^56^ Briefly, samples were classified based on DNA copy number, mRNA and microRNA transcriptomics, methylomics and proteomics analyses of the patient cohort.^56^ Expression levels of *CHEK1* and *BRD4* across different subtypes of HCC were highly varied and only sample from iCluster2 exhibited lower expression of *CHEK1* (Figure 4a). Interestingly, iCluster2 samples were clinically associated with low‐grade tumors and less microvascular invasion, and associated with the nonproliferative subclass of HCC.^56^, ^57^ On the other hand, iCluster1 and iCluster3 samples are associated with the proliferative subclass, characterized by an activation of the cell cycle signaling pathway.^57^ We then proceeded to determine whether expression levels of *CHEK1* and *BRD4* were differently expressed in HCC patients when stratified according to the expression levels of *MYC*. Stratification by *MYC* levels did not correlate with any significant differences in *CHEK1* and *BRD4* expression, suggesting that *CHEK1* and *BRD4* expression is diverse in HCC patients and independent of *MYC* expression (Figure 4b).

Given the highly varied expression levels of *CHEK1* and *BRD4* observed in the TCGA‐LIHC cohort, survival analyses were then performed to determine if the varied expression of *CHEK1* and *BRD4* conferred any survival benefit to HCC patients. We found that stratification of patients solely on *MYC* expression did not confer significant survival benefits (Figure 4ci). However, *MYC* expression contributes to the development and progression of aggressive HCC as there was a greater distribution of patients with higher grade tumors (neoplasm histologic grade 3 or 4) in the MYC^Hi^ group versus the MYC^Lo^ group (42.62% vs. 31.18%) (Figure 4cii,iii). When patients were further stratified based on the *CHEK1* and *BRD4* expression levels, the data revealed that low expression of all three targets conferred greater survival benefit for HCC patients (Figure 4di). Consistent with the role of MYC, CHK1 and BRD4 in promoting aggressive HCC, higher‐grade tumors were observed in patients with high expression of all three targets as opposed to those with low expression levels (55.56% vs. 18.00%) (Figure 4dii,iii). Importantly, expression levels of *CHEK1* and *BRD4* did not confer significant survival benefit in patients with MYC^Lo^ tumors (Figure 4e). However, there was a significant correlation between overall patient survival and *CHEK1*/*BRD4* expression in the MYC^Hi^ group as patients with high expressions of *CHEK1* and *BRD4* exhibited poorer prognosis compared to those with low expression levels, reflecting a specific dependency on CHK1 and BRD4 only in MYC‐deregulated tumors (Figure 4f). Taken together, the data suggest the role of CHK1 and BRD4 in contributing to the oncogenic capacities of MYC in the pathogenesis of aggressive MYC‐driven HCC. Notably, the data suggest that MYC synthetic lethality is not necessarily contingent on simultaneous overexpression of both targets but rather, endogenous levels of molecular nodes such as BRD4 and CHK1 become critical to the survival of MYC‐addicted cancer cells.

### 2.5 Targeting CHK1 and BRD4 in human HCC patient‐derived avatars

**FIGURE 5:**
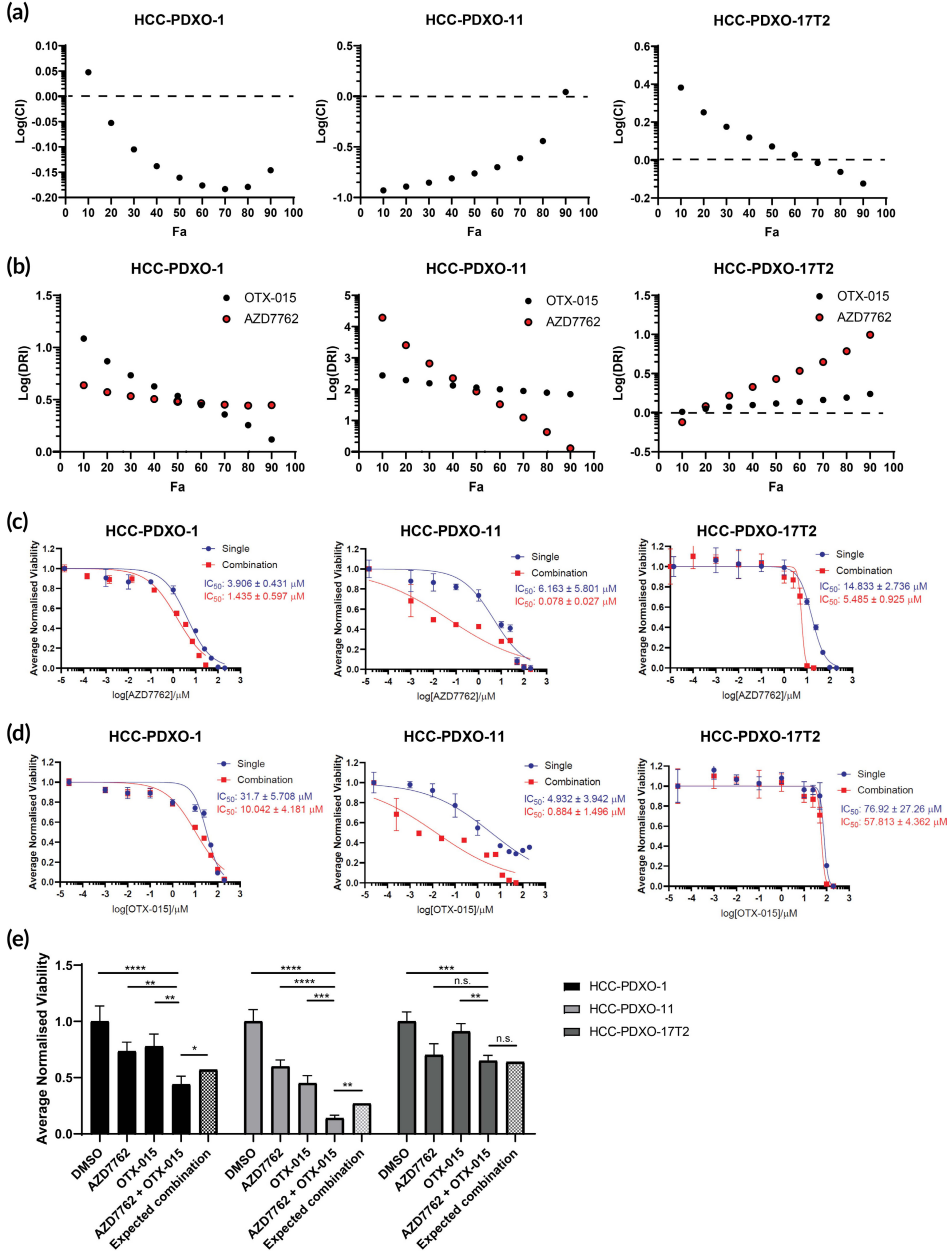
Pharmacological inhibition of CHK1 and BRD4 is a synergistic combination against MYC‐driven HCC. (a) F_a_‐combination indices plot and (b) F_a_‐dose reduction indices plot of CHK1 inhibitor, AZD7762, and BRD4 inhibitor, OTX‐015 in MYC^Hi^ HCC‐PDXO‐1, HCC‐PDXO‐11, and MYC^Lo^ HCC‐PDXO‐17T2 as calculated based on the Chou‐Talalay method. Indices are represented as means of three biological repeats. Monotherapy versus combination therapy dose–response curves and IC_50_ analysis of (c) AZD7762 and (d) OTX‐015 in HCC‐PDXO‐1, HCC‐PDXO‐11, and HCC‐PDXO‐17T2. IC_50_ values are represented as means ± SD (*n* = 3). (e) Bliss‐independence validation of AZD7762 and OTX‐015 in HCC‐PDXOs as quantified by CTG. Expected viabilities of HCC‐PDXOs are the product of the singlet viabilities. Statistical analyses between treatment groups were performed using ordinary one‐way ANOVA and Dunnett's pairwise comparisons as recommended. Statistical analyses between observed and expected viabilities following combination therapy were performed using one‐sample *t*‐test. (n.s.: not significant; *p < 0.05; **p < 0.01; ***p < 0.001).

We evaluated the efficacy of CHK1 and BRD4 small‐molecule inhibitors, AZD7762 and OTX‐015, respectively, in HCC patient‐derived xenograft organoid (HCC‐PDXO) models. We calculated the combination index (CI) via the Chou‐Talalay method in three HCC‐PDXO lines (two MYC^Hi^ and one MYC^Lo^) to determine whether the pair of drugs exhibit a synergistic (CI < 1; LogCI < 0), additive (CI = 1; LogCI = 0), or antagonistic (CI > 1; LogCI > 0) relationship (Figure S3A).^58^, ^59^ The IC_50_ values of both drugs were first established via dose–response viability assays, and the ratio of the IC_50_ values was used in the derivation of the CIs (Figure S3B). The CIs across the spectrum of factor of cells affected (F_a_) were reflective of a synergistic relationship (LogCI < 0) between AZD7762 and OTX‐015 in MYC^Hi^ HCC‐PDXO lines, HCC‐PDXO‐1, and HCC‐PDXO‐11 (Figure 5a). Conversely, the combination was largely antagonistic (LogCI > 0) in MYC^Lo^ HCC‐PDXO line, HCC‐PDXO‐17T2, except for the upper F_a_ range. Further interrogation into the dose‐reduction indexes (DRI) of both drugs in the HCC‐PDXOs demonstrated the synergy of the drug combination only in MYC^Hi^ HCC‐PDXOs. The DRI reflects the extent of dose reduction observed for a single drug when used in combination to achieve the same effect to when it is administered singly.^58^, ^59^ Expectedly, a favorable dose reduction (DRI > 1; logDRI > 0) and corresponding marked leftward shift in the dose–response curves was observed for both drugs in both MYC^Hi^ HCC‐PDXOs, while only AZD7762 exhibited a favorable DRI and reduction in IC_50_ in HCC‐PDXO‐17T2 at the higher F_a_ range (Figure 5b–d). The dose reduction in OTX‐015 was insufficient to elicit a synergy between the drugs in MYC^Lo^ HCC‐PDXO‐17T2 (Figure 5b,d).

We additionally confirmed the unique synergy of AZD7764 and OTX‐015 in MYC‐deregulated HCC via the bliss independence model. A synergistic drug interaction is defined as one when the observed viability of the cells in vitro is significantly lower than its expected viability—the product of its singlet viabilities.^60^ Treatment with AZD7762 and OTX‐015 singly and in combination indicated a similar trend in the HCC‐PDXOs to the CIs and DRIs as both MYC^Hi^ HCC‐PDXO‐1 and HCC‐PDXO‐11 had a significant reduction in the organoid viability in vitro than expected, more so for HCC‐PDXO‐11 (Figure 5e). On the other hand, the viability of HCC‐PDXO‐17T2 was comparable to its expected viability when treated with the drug combination, demonstrating the limited efficacy of the combination in MYC^Lo^ lines (Figure 5e).

### 2.6 AZD7762 and OTX‐015 combination therapy mitigates cancer growth in HCC organoids

**FIGURE 6:**
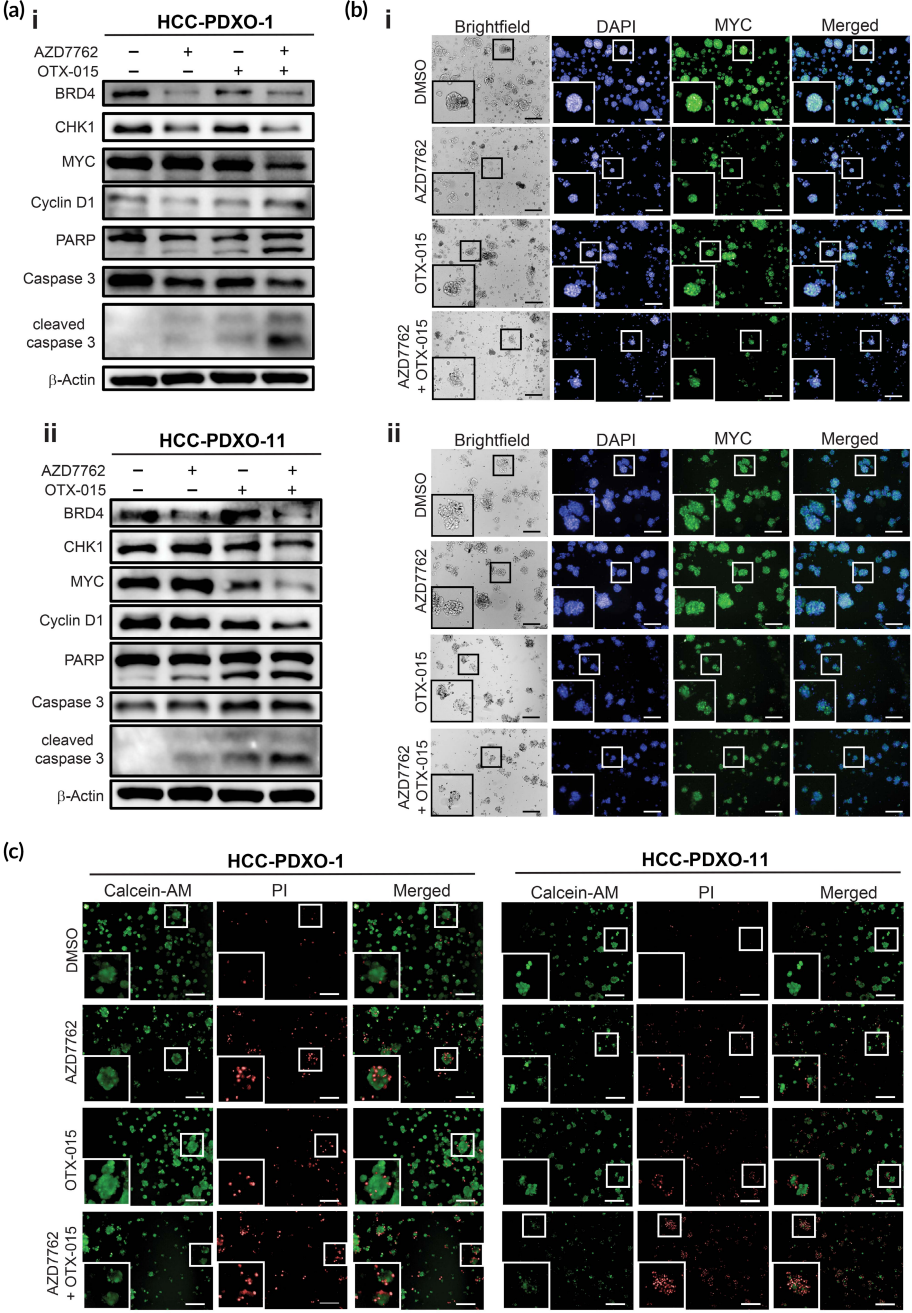
AZD7762 and OTX‐015 combination therapy disrupts organoid growth in HCC‐PDXOs in vitro. (a) Immunoblots of MYC and apoptosis markers in (i) HCC‐PDXO‐1 and (ii) HCC‐PDXO‐11 following treatment with AZD7762 and OTX‐015. (b) Immunofluorescence of MYC expression levels and brightfield images of (i) HCC‐PDXO‐1 and (ii) HCC‐PDXO‐11 after combination therapy of AZD7762 and OTX‐015 (scale bar, 200 μM). (c) Live/dead cell viability assay of HCC‐PDXO‐1 and HCC‐PDXO‐11. HCC‐PDXOs were stained with fluorescence markers calcein‐AM and propidium iodide (scale bar, 200 μM). Fluorescence and brightfield images were taken with the Operetta high‐content screening microscope and insert images were magnified by a factor of 2.5.

Finally, we explored the functional effects of the SSO‐QPOP‐identified combination in HCC‐PDXO‐1 and HCC‐PDXO‐11. Combination treatment with AZD7762 and OTX‐015 caused a significant reduction in MYC expression levels in both HCC‐PDXO as compared to when they were singly treated, as with the SSOs (Figure 6a). Additionally, expression levels of apoptosis markers were correspondingly elevated in the HCC‐PDXOs treated with the combination, demonstrating the potential of AZD7762 and OTX‐015 against MYC‐deregulated HCC (Figure 6a). Immunofluorescence was also performed to visualize the effects of the combination on organoid morphology and viability in vitro. Similarly, combinatorial treatment led to a downregulation of MYC expression levels (Figure 6b, S4A). Furthermore, combination therapy with AZD7762 and OTX‐015 attenuated the formation and growth of the HCC‐PDXOs, evidenced by the significant decrease in organoid size, as well as disruption of the dense spherical structures which are typically characteristic of viable organoids (Figure 6b, S4B). Organoids were stained with calcein‐AM dye and propidium iodide to determine the ratio of dead to live cells in the HCC‐PDXOs posttreatment. The ratio of dead to live cells in the HCC‐PDXOs co‐treated with both drugs were significantly higher compared to the monotherapies, indicative of enhanced cell killing in vitro (Figures 6c, S4C). The results collectively demonstrated that AZD7762 and OTX‐015 can synergistically suppress MYC levels and induce cell death in patient‐derived HCC models, highlighting the clinical value of concurrently inhibiting CHK1 and BRD4 in MYC‐deregulated HCC.

## 3 DISCUSSION

Synthetic lethality offers an alternative therapeutic strategy against tumors harboring a dependency on deregulated oncogenes which are otherwise undruggable, such as MYC. MYC deregulation has been identified as a key oncogenic driver in up to 60% of HCC cases, the predominant form of liver cancer.^61^ In HCC, numerous synthetic lethal targets of MYC have been previously identified, such as BRD4, CDK9, and aurora kinase A, among others.^3^, ^11^ However, there are no drugs against MYC synthetic lethality which have been approved for the treatment of MYC‐driven HCC despite having progressed into clinical trials.^9^ There is therefore a need to prioritize synthetic lethal targets of MYC so that greater efforts can be channeled into the development of therapeutics with greater clinical prospects and tolerability.

Here, we demonstrate the capability of an experimental–analytical hybrid platform, SSO‐QPOP, to prioritize gene targets based on their therapeutic capacities. QPOP is conventionally used to rank drug combinations based on their overall efficacies and can therefore prioritize which drugs have the greatest therapeutic potential amongst a panel of drugs.^28^, ^62^, ^63^ Given that drugs may have nonspecific and off‐target effects, replacing drug panels in QPOP with emerging RNA therapeutics such as antisense oligonucleotides can therefore be used to prioritize specific targets. Here, we employed SSOs as our choice of genetic modulator as they have been shown to exhibit a dose‐dependent effect on their target genes and are promising candidate therapeutics.^38^, ^48^, ^64^ In this iteration of SSO‐QPOP, we selected four SSOs against MYC synthetic lethal targets (*CHEK1*, *MAX*, *MCL1*, and *BRD4*) with the greatest knockdown efficiencies amongst a panel of eight targets, together with two HCC standard‐of‐care drugs, sorafenib and cabozantinib to compare the efficacy of targeting MYC synthetic lethality with the standard‐of‐care drugs.^65^, ^66^

Based on the iterations of QPOP analyses performed on HCC cell lines stratified based on their levels of MYC expression, CHK1 and BRD4 have been identified as the most promising target combination against MYC‐driven HCC as simultaneous functional knockout of both targets were among the top‐ranking combinations in only MYC^Hi^ cell lines. Normalizing the results from MYC^Hi^ cell lines to MYC^Lo^ cell lines, provides additional support that concurrent ablation of CHK1 and BRD4 function induces the greatest reduction in viability of MYC^Hi^ cell lines while minimizing any off‐target effects on cells with lower MYC expression. These results support the notion that the CHK1‐BRD4 combination is likely to exhibit minimal toxicities in normal cells with lower MYC expression levels due to the presence of functioning cell cycle regulation mechanisms. Notably, the ssCHK1 and ssBRD4 combination is identified only from a panel of four targets. Hence, it is possible that other MYC synthetic lethal targets not evaluated in this study are more effective. However, it is challenging to simultaneously knockdown many genes due to the limited transfection efficiency of the transfection agents. Moreover, toxicity due to physiological limits in drug clearance reduces the therapeutic index of each drug as the number of drugs administered is increased. Future iterations of SSO‐QPOP can therefore be performed in a systematic manner by sequentially replacing ineffective targets with alternative MYC synthetic lethal targets until the best target is identified. In doing so, a broader range of synthetic lethal targets can be evaluated and prioritized, and the SSO‐QPOP platform could be potentially useful to discover subsequent target combinations addressing drug resistance. Additionally, various molecular mechanisms underpinning MYC deregulation in HCC have been reported, including *MYC* amplification, point mutations promoting MYC stability, and β‐catenin pathway activation.^67^, ^68^, ^69^, ^70^, ^71^, ^72^ The heterogeneous landscape of MYC addition would therefore confer differential sensitivities to SSOs targeting regulators of *MYC* expression, synthetic lethal targets in pathways downstream of MYC activation, and components in the β‐catenin pathway respectively.^9^ Characterization of MYC‐deregulated tumors can therefore streamline the SSO‐QPOP pipeline further as an optimized panel of effective SSOs can be curated according to the mechanisms of MYC deregulation.

Our molecular analysis suggests that the simultaneous attenuation of both CHK1 and BRD4 works to impede MYC‐induced oncogenesis via two mechanisms. First, ssCHK1 and ssBRD4 hindered G1/S transition cell cycle progression by depleting MYC and cyclin D1 levels, while simultaneously upregulating tumor suppressor genes in HCC, preventing cell growth in target cells.^52^, ^73^, ^74^, ^75^, ^76^, ^77^, ^78^ Second, ssCHK1 and ssBRD4 co‐treatment resulted in an incompetent DNA damage coping mechanism, leading to an accumulation of DNA damage beyond tolerable levels, thereby inducing apoptosis in target cells. Additionally, previous studies have demonstrated that MYC‐deregulated tumors depend on DNA damage regulator CHK1 or BRD4 to manage DNA damage arising from MYC‐induced replicative stress, sensitizing them to CHK1 or BRD4 inhibition.^16^, ^54^, ^55^, ^79^ Our collective data therefore suggest that ssCHK1 and ssBRD4 exhibit anticancer properties in combination in MYC‐driven cancers and have the potential to serve as therapeutic agents following subsequent downstream optimization for in vivo delivery.

As CHK1 and BRD4 are known targets to have pharmacological inhibitors which have proceeded to clinical trials, we also validated the efficacy of using these inhibitors in combination in PDXO models of HCC. HCC‐PDXOs have been shown to recapitulate the native tumor microenvironment, heterogeneity, and architecture in vitro, highlighting the clinical relevance HCC‐PDXOs as in vitro models of the disease.^80^, ^81^, ^82^ Validation of CHK1 and BRD4 inhibitors, AZD7762 and OTX‐015, respectively, in three HCC‐PDXO lines have demonstrated that this combination exhibits synergistic interactions only in MYC^Hi^ HCC‐PDXOs. This mirrors SSO‐QPOP analyses where the genetic ablation of CHK1 and BRD4 was identified as the most effective combination specific to MYC‐deregulated HCC, highlighting the translatability of SSO‐QPOP target prioritization in identifying clinically actionable targets and combinations. Considering this, we propose that SSO‐QPOP can guide the co‐development of effective cancer therapeutics, such as CHK1 and BRD4 inhibitors against MYC‐driven HCC.

However, the progress of both AZD7762 and OTX‐015 in the clinics has been terminated due to cardiac dose‐limiting toxicities and a lack of clinical efficacy, respectively (NCT00413686, NCT02698176, NCT02296476, NCT02698189).^83^ Furthermore, developing pharmacological agents against BRD4 is challenging as the acetyl‐lysine binding pockets of the inhibitors are highly conserved across the bromodomain and extraterminal family of proteins, resulting in limited selectivity of the inhibitors toward BRD4 and adverse dose‐limiting toxicities in the clinical trials, such as for molibresib.^84^, ^85^, ^86^ This has led to the suspension of the development of BRD4 inhibitors in the clinics, such as BAY1238097.^87^ There is, therefore, a need to develop alternative therapeutics against CHK1 and BRD4 with better clinical prospects. Notably, we demonstrated the effectiveness of SSOs against both CHK1 and BRD4 in targeting MYC‐driven HCC cells. Prior studies have also exhibited the value of SSOs as candidate therapeutic agents in cancer.^38^, ^45^, ^46^, ^47^, ^88^ It may therefore be of value to further develop and optimize ssCHK1 and ssBRD4 as potential therapeutic agents against MYC‐driven HCC in a situation where no viable pharmacological agents are available for clinical use. Further chemical modifications to ssCHK1 and ssBRD4 such as the use of a morpholino‐based backbone can render them resistant to enzymatic degradation in biologic fluids and suitable for subsequent clinical applications, as demonstrated in the FDA‐approved SSOs.^40^, ^41^, ^42^, ^43^ Upon chemical optimization of ssCHK1 and ssBRD4 and in vivo validations, clinical trials in HCC patients could be initiated to investigate the therapeutic value of ssCHK1 and ssBRD4 in combination. Furthermore, inhibitors against CHK1 and BRD4 are currently trialed in combination with immunotherapies in patients with advanced solid tumors, including HCC (NCT04840589, NCT03059147, NCT03495323). Similar trials with ssCHK1 and ssBRD4 in combination with anti‐PD‐1/PD‐L1 immunotherapies may also be considered. It is worth mentioning that while SSO‐QPOP can identify targets with the greatest the clinical value specific to MYC‐deregulated tumors, it is necessary to stratify patients according to the level of MYC deregulation as demonstrated in prior clinical studies.^9^, ^89^ Based on the QPOP analyses, concurrent targeting of CHK1 and BRD4 is effective in MYC^Hi^ tumors, while exhibiting limited efficacy in MYC^Lo^ lines. Hence, it is necessary to stratify patients according to their level of deregulation and dependency on MYC as an oncogenic driver prior to clinical evaluation of CHK1 and BRD4 combination therapies.

Finally, the data presented in this study point toward the potential application of the SSO‐QPOP pipeline in the clinical setting to prioritize therapeutic targets and guide cancer therapies. Ex vivo SSO‐QPOP can be performed on patient samples using a predetermined panel of SSOs curated based on the molecular characterization of the patient tumor sample to rapidly determine the optimized combination with the best therapeutic index for the patient. For example, patients with MYC deregulation can be screen with a panel of SSOs against synthetic lethal targets of MYC, while patients with mutations in *CTNNB1*, a frequent genetic alteration in HCC, can be screened with a panel of SSOs targeting the β‐catenin pathway.^90^ Upon identification of an effective combination, patients can be treated with FDA‐approved drugs against the prioritized targets if available, and cancer‐specific biomarkers can be used to evaluate the optimal dose and corresponding efficacy of a drug combination in patients. A prior study has used the patient's serum level of prostate‐specific antigen (PSA) as a biomarker for the patient response to the treatment.^91^ HCC‐specific biomarkers, such as α‐fetoprotein, and serial imaging of liver tumor nodules can therefore be leveraged on to assess and determine the therapeutic efficacy of the SSO‐QPOP‐identified combination.^90^, ^92^, ^93^ With a sufficiently large patient cohort in long run, clinical responses of all the patients can then be aggregated and used for a more robust QPOP analyses for clinical treatments.

## 4 CONCLUSION

To conclude, we demonstrate a proof‐of‐concept that given a panel of genetic modulators, SSO‐QPOP can prioritize MYC synthetic lethal targets with the greatest therapeutic potential and compare their therapeutic value to the current standard‐of‐care drugs. More importantly, validation of the SSO‐QPOP‐identified combination of targeting CHK1 and BRD4 in our clinically relevant HCC‐PDXO models demonstrates the strong synergy and clinical actionability of the combination when HCC‐PDXOs are stratified based on their levels of MYC deregulation. We therefore suggest that concurrent inhibition of CHK1 and BRD4, as prioritized by SSO‐QPOP, is a promising treatment approach for MYC‐driven HCC, and that ssCHK1 and ssBRD4 can serve as promising candidate therapeutic agents following subsequent optimization.

## 5 MATERIALS AND METHODS

### 5.1 Patient‐derived xenograft establishment and organoid culture

Primary patient tumor samples were obtained with consent from the livers of HCC patients at the National University Hospital, Singapore General Hospital and National Cancer Centre Singapore. Tissue samples were minced into fine pieces and mixed 1:1 (v/v) with Matrigel™. The tissue mixtures were then introduced into immunocompromised triple transgenic nonobese diabetic (NOD) scid gamma (NSG) mice via subcutaneous injection into the left flank to establish the HCC‐PDXs. Serial transplantation of the HCC‐PDXs was performed similarly when the tumor volumes reach 2000 mm^3^. All protocols for animal studies were reviewed and approved by the National University of Singapore Institutional Animal Care and Use Committee (IACUC). To establish HCC‐PDXO cultures, HCC‐PDX tumors were extracted and processed as described previously.^80^ Briefly, the tumors were minced and incubated in 37°C with 1 mg/ml collagenase/dispase® for 30 min. Fragmented tumor cells were subsequently washed with Dulbecco's Modified Eagle Medium (DMEM) supplemented with 5% penicillin/streptomycin before being passed through a 100 μm cell strainer and harvested via centrifugation. HCC‐PDXOs were established by resuspending the dissociated tumor cells in DMEM/F‐12 and mixed 1:4 (v/v) with Matrigel™ before seeding into 24‐well plates. The HCC‐PDXOs were cultured in DMEM/F‐12 supplemented with the necessary growth factors as described previously.^94^

### 5.2 SSO design

SSOs were synthesized as single‐stranded antisense oligonucleotides with 2′‐O‐methyl‐modified RNA bases linked by a phosphorothioate backbone by Genscript Biotech Corp (Hong Kong). SSOs were rationally designed for optimal efficiency in inducing splicing events at target sites by exerting steric hindrance against splicing factors, as previously described.^36^, ^37^ Briefly, SSOs were designed by a computational algorithm which account for: (1) co‐transcriptional binding accessibilities, (2) binding thermodynamics, and (3) presence of regulatory splicing motifs.

### 5.3 QPOP model generation

HCC cell lines were first reverse transfected with the SSOs component of the 50 experimental combinations necessary for sufficient factor screening and in‐depth analyses based on the OACD (Table S2).^33^ ssNeg negative control SSO was used to ensure that the total SSO concentration was kept at a constant of 200 nM for all combinations. Twenty‐four hours after reverse transfection, the cells were treated with the respective sorafenib and cabozantinib components of the 50 combinations for 48 h before their viability were quantified using the CellTiter 96®AQ_ueous_ nonradioactive cell proliferation MTS assay as the phenotypic input for QPOP (Tables S1 and S2).

The viability of the HCC cell lines was fitted into a second‐order quadratic series as shown as follows:

$$y=\alpha +\beta_{1}x_{1}+\ldots +\beta_{j}x_{j}+\beta_{12}x_{1}x_{2}+\ldots +\beta_{\mathrm{ij}}x_{i}x_{j}+\beta_{11}{x_{1}}^{2}+\ldots +\beta_{\mathrm{jj}}{x_{j}}^{2}$$

where *y* is the desired model output, *α* is the intercept, *x*_*j*_ is the *j*th drug dose, *β*_*j*_ is the coefficient for the single‐drug dose of the *j*th drug, *β*_*ij*_ is the coefficient for the interaction between the *i*th and *j*th drug, and *β*_*jj*_ is the quadratic coefficient for the *j*th drug. MATLAB was used to perform a stepwise regression analysis with the second‐order quadratic function to project the expected cell viabilities for all possible combinations as the output. The parabolic response surface maps were also generated with MATLAB.

Additional methods are described in Supplementary Material and Methods S1.

## AUTHOR CONTRIBUTIONS

**Dexter Kai Hao Thng:** Conceptualization (equal); data curation (lead); formal analysis (lead); investigation (lead); methodology (equal); validation (lead); visualization (lead); writing – original draft (lead); writing – review and editing (equal). **Tan Boon Toh:** Conceptualization (equal); data curation (supporting); formal analysis (supporting); methodology (equal); resources (equal); visualization (equal); writing – review and editing (equal). **Paolo Pigini:** Conceptualization (equal); formal analysis (supporting); methodology (equal); resources (equal); writing – review and editing (equal). **Lissa Hooi:** Project administration (equal); resources (equal). **Yock Young Dan:** Resources (supporting). **Pierce Kah‐Hoe Chow:** Resources (supporting). **Glenn Kunnath Bonney:** Resources (supporting). **Masturah Bte Mohd Abdul Rashid:** Conceptualization (supporting); resources (supporting); software (equal). **Ernesto Guccione:** Conceptualization (supporting). **Dave Keng Boon Wee:** Conceptualization (equal); formal analysis (supporting); funding acquisition (equal); methodology (equal); resources (equal); writing – review and editing (equal).

## CONFLICT OF INTEREST

Edward Kai‐Hua Chow is a shareholder in KYAN Therapeutics.

### PEER REVIEW

The peer review history for this article is available at https://publons.com/publon/10.1002/btm2.10363.

## Supporting information


Appendix S1 Supporting Information

Table S1 QPOP combination design using orthogonal array composite design consisting of 50 combinations for six modulators at three dosages (−1, 0, 1).
**Table S2. Concentrations of sorafenib and cabozantinib used for QPOP in Bel7402, HCCLM3 and SNU387 at 3 dosages (−1, 0 1).** IC_15_ and IC_30_ values are used for QPOP if the IC_50_ value is lower than C_max_. Else, 5% and 10% C_max_ values are used for QPOP. IC_50_ values are represented as means ± SD (n = 3).
**Table S3. Parameter estimates and significance of QPOP analyses on Bel7402, HCCLM3 and SNU398.** Statistical analyses were performed using sum of squares F‐test (*p < 0.05; **p < 0.01; ***p < 0.001).
**Table S4. Top‐ranked two‐modulator combinations for MYC** ^**Hi**^ **Bel7402 and HCCLM3 when normalized to MYC** ^**Lo**^ **SNU387.** Overall rankings are in parentheses.
Table S5. List of primer sequences and antibodies.

## Data Availability

The data that support the findings of this study are available from the corresponding author upon reasonable request. Data pertaining to the generation of the QPOP model is not available as itis proprietary to KYAN Therapeutics. SSO sequences are undergoing patent application.
