# Supplementary material for: Splice‐switch oligonucleotide‐based combinatorial platform prioritizes synthetic lethal targets CHK1 and BRD4 against MYC‐driven hepatocellular carcinoma
Source: Bioeng Transl Med. 2022 Sep 3;8(1):e10363. doi: 10.1002/btm2.10363 (PMC9842033; doi:10.1002/btm2.10363)
Supplement: Supplementary file 1 — Appendix S1 Supporting Information [file BTM2-8-e10363-s002.zip › DKHT_BioTM_SI.docx]

**Supplementary Information for**

Splice-switch oligonucleotide-based combinatorial platform prioritises synthetic lethal targets CHK1 & BRD4 against MYC-driven HCC

**This PDF file includes:**

Supplementary Materials and Methods

Supplementary Figure Legends

**Supplementary Materials and Methods**

*Cell culture*

Hepatocellular carcinoma (HCC) cell line, Bel7402 and HCCLM3 were cultured in high glucose Dulbecco’s Modified Eagle’s medium (DMEM) supplemented with 10% fetal bovine serum, 5% MEM non-essential amino acids and 5% penicillin/streptomycin. SNU387 was cultured in Roswell Park Memorial Institute (RPMI) 1640 medium supplemented with 10% fetal bovine serum, 5% sodium pyruvate and 5% penicillin/streptomycin. All cell lines were maintained in a 95% humidified atmosphere of 5% CO_2_ at 37°C.

*SSO validation*

Induction of splicing events by SSOs were validated via quantitative real time-polymerase chain reaction (qRT-PCR) and gel electrophoresis. For qRT-PCR protocol (including total RNA extraction and reverse transcription), refer to the subsequent relevant section. For gel electrophoresis, 20ng of cDNA is first amplified using the GoTaq® PCR Core System as per manufacturer’s instructions. Primers were designed to flank the skipped exons so that the splicing efficiency can be detected by the band shift of the PCR amplicon. PCR amplicons were resolved on a 2.5% agarose gel and imaged using the Gel Doc™ XR+ imager. Primer sequences are detailed in Table S5.

*SSO* *transfection*

Bel7402 cells were transiently transfected with the SSOs with transfection reagent Lipofectamine® RNAiMAX. Briefly, SSOs were mixed with Lipofectamine® RNAiMAX in Opti-MEM® reduced serum medium for 20 minutes before the Bel7402 cells were transfected with the reaction mixture at a final concentration of 1 or 50nM of SSOs and a dilution factor of x333 of Lipofectamine® RNAiMAX. Cells were transfected with SSOs for a duration of 48 hours before processing for downstream analyses.

*Quantitative real time polymerase chain reaction (qRT-PCR)*

Total RNA content were extracted using the RNeasy® Plus Mini Kit as per manufacturer’s instructions. 1μg of RNA was then reverse transcribed with iScript™ Reverse Transcription Supermix as per manufacturer’s protocol. 20ng of cDNA per sample per gene were subject to 40 cycles of amplification with iTaq™ SYBR® Green Supermix in the Applied Biosystems QuantStudio 5 Real-Time PCR System. PCR reactions contained 5µL of iTaq™ SYBR® Green Supermix (2x), 0.4µL of forward and reverse primer mix (final concentration of 200nM), 2µL of cDNA (20ng) and topped up with nuclease-free water to a total volume of 10µL. The sequences of primers used, and their applications are detailed in Table S5.

*Immunoblotting*

Whole cell lysates were extracted from cell pellets with a radioimmunoprecipitation assay (RIPA) buffer cocktail, comprising of 10mM Tris-Hcl (pH 7.4), 1% NP40, 0.50% sodium deoxycholate, 150mM sodium chloride, 0.10% sodium dodecyl sulfate, and Pierce™ protease and phosphatase inhibitor cocktail tablets. The lysates were resolved on 10% or 12% polyacrylamide gels and transferred onto a poyvinylidene fluoride (PVDF) membrane before being probed with primary and secondary antibodies. The chemiluminescence signal was visualised on the ChemiDoc™ Imaging System and the relative band intensities were quantified with the Bio-Rad Image Lab software. Antibodies used are listed in Table S5.

*Drug treatment*

HCC cell lines and HCC-PDXOs were seeded for 24 hours prior to any drug treatment. 48 hours after drug treatment, the cells were either pelleted or subjected to the respective viability assays for the determination of the half-maximal inhibitory concentration (IC_50_) and bliss independent model validations. Dose-response viability assays were performed in 384-well plates and cells were treated with log dose concentrations of the drugs. HCC cell line viability was determined using the absorbance readout of the CellTiter 96®AQ_ueous_ non-radioactive cell proliferation MTS assay, whilst the viability of HCC-PDXOs was measured using the luminescence readout from the CellTitre-Glo® (CTG) assay. GraphPad Prism software was used to establish the sigmoidal dose-response curves and respective IC_50_ values for each drug and line.

*Flow cytometry analysis*

Level of apoptosis in singly and co-transfected cells were determined with the BD Pharmingen™ Fluorescin Isothiocynate (FITC) Annexin V apoptosis detection kit as per manufacturer’s instructions. Briefly, transfected cells were harvested and resuspended in Annexin V binding buffer containing 10µL of Annexin V-FITC and 5µL of propidium iodide (PI) (50µg/mL). The cells were subsequently incubated in the dark for 15 minutes and subjected to flow cytometry analysis using the BD LSR II flow cytometer. Quantification of FITC and PI intensities were performed on the FlowJo software.

*Immunofluorescence*

Pre-treated organoids in 96-well plates were fixed in 4% paraformaldehyde for 30 minutes before permeabilization with 0.5% triton X-100 for 30min. After permeabilization, the organoids are incubated with the blocking buffer comprising of 10% normal horse serum, 0.1% bovine serum albumin and 0.2% triton X-100 for 2 hours. Following which, MYC primary antibody was added to the organoids overnight at room temperature. Excess primary antibody was removed by washing the organoids thrice with DPBS supplemented with 0.1% Tween-20 (DPBS-T). The organoids were then incubated with secondary antibody and DAPI for 2 hours before being washed thrice with DPBS-T and imaged. Antibodies used are listed in Table S5.

Calcein-AM and PI were used to co-stain the HCC-PDXOs to determine the presence of live and dead cells in the pre-treated organoids seeded in 96-well plates. Hoechst 33342 was used to stain the nucleus of the organoids. 48 hours after drug treatment, the organoid medium was aspirated and replaced with 75uL of staining solution containing calcein-AM (final concentration of 4 mM), PI (final concentration of 10 mg/ml), and Hoechst 33342 (final concentration of 10 mg/ml) for 1 hour of incubation prior to imaging.

All organoid fluorescence images were acquired using the Operetta high-content screening microscope and fluorescence intensity quantified using the Harmony® high-content imaging and analysis software.

*Statistical analyses*

Statistical analyses were conducted on the GraphPad Prism software. Comparisons within groups were performed using ordinary one-way ANOVA and the respective pairwise comparison as recommended by the software at a 5% significance level. One-sample t-test was used to determine the statistical different between the expected and observed viability of the cells in the bliss independence model at a 5% significance level. Survival analyses were performed using the Gehan-Breslow-Wilcoxon test at a 5% significance level. Expression levels of *MYC*, *CHEK1* and *BRD4* from the GDC TCGA LIHC database were normalised to their respective z-score and samples were stratified into two groups – low- and high-expressing groups at the 50^th^ percentile. Statistical analyses of the QPOP regression model were performed on MATLAB together with the establishment of the model. All data shown are means and standard deviations of three biological replicates, unless otherwise stated.

**Supplementary Figure Legends**

**Figure S1. SSO design and characterisation for QPOP application.** (A) Schematics of target gene mRNAs. Target sites of SSOs are indicated with arrows, detailing exons skipped and functional domains affected by the splice-switch. Schematics are obtained from SPLICEMOD™ on TechNOA. Relative expression of (B) spliced and (C) total transcripts of target genes following SSO transfection at 1nM and 50nM. Ordinary one-way ANOVA and Tukey’s pairwise comparisons were performed as recommended (n.s.: not significant; *p < 0.05; **p < 0.01; ***p < 0.001). (D) Quantification of the relative protein expression levels of target genes post-transfection in the presence of proteasome inhibitor MG132, from Figure 1D. Ordinary one-way ANOVA was performed to determine statistical differences within the group (n.s.: not significant; *p < 0.05; **p < 0.01; ***p < 0.001). (E) Optimising experimental conditions for QPOP. (*Left*) Normalised viability of Bel7402 24 hours after co-transfection with four different SSOs (ssCHK1, ssMAX, ssMCL1 and ssBRD4) at 50nM each. (*Right*) Relative expression of spliced transcripts of SSO targets genes at 4, 8, 12 and 24 hours post-transfection. Multiple unpaired t-tests were performed and corrected using the Holm-Sidak method as recommended (n.s.: not significant; *p < 0.05; **p < 0.01; ***p < 0.001).

Figure S2. Validation of QPOP analyses in MYC^Hi^ Bel7402. (A) Immunoblot of relative MYC expression levels in seven HCC cell lines. Bel7402 and HCCLM3 are representative MYC^Hi^ cell lines, and SNU387 and Huh7 are representative MYC^Lo^ cell lines. (B) Parabolic response surface maps of poorly-ranked combinations (ssMAX with (*Left*) ssMCL1 and (*Right*) ssCHK1) in Bel7402. (C) Validation of poorly-ranked combinations in Bel7402 as quantified by MTS. Ordinary one-way ANOVA and Dunnett’s pairwise comparisons were performed as recommended (n.s.: not significant; *p < 0.05; **p < 0.01; ***p < 0.001).

Figure S3. Characterisation of MYC^Hi^ and MYC^Lo^ HCC-PDXOs. (A) Immunoblot of relative MYC expression levels in a panel of 14 HCC-PDXs. HCC-PDXO-1 and HCC-PDXO-11 are MYC^Hi^ while HCC-PDXO-17T2 is MYC^Lo^. (B) Representative dose-responses of HCC-PDXO-1, HCC-PDXO-11 and HCC-PDXO-17T2 to AZD7762 and OTX-015. IC_50_ values are represented as means ± SD (n = 3).

Figure S4. Quantification of immunofluorescence images in Figure 6. Quantification of (A) MYC fluorescence intensity levels, (B) organoid size, and (C) ratio of total propidium iodide intensity to total calcein-AM intensity per well using the Harmony® high-content imaging and analysis software. Ordinary one-way ANOVA and Dunnett’s pairwise comparisons were performed as recommended (n.s.: not significant; *p < 0.05; **p < 0.01; ***p < 0.001).
