# Supplementary figures and images for: Splice‐switch oligonucleotide‐based combinatorial platform prioritizes synthetic lethal targets CHK1 and BRD4 against MYC‐driven hepatocellular carcinoma
Source: Bioeng Transl Med. 2022 Sep 3;8(1):e10363. doi: 10.1002/btm2.10363 (PMC9842033; doi:10.1002/btm2.10363)

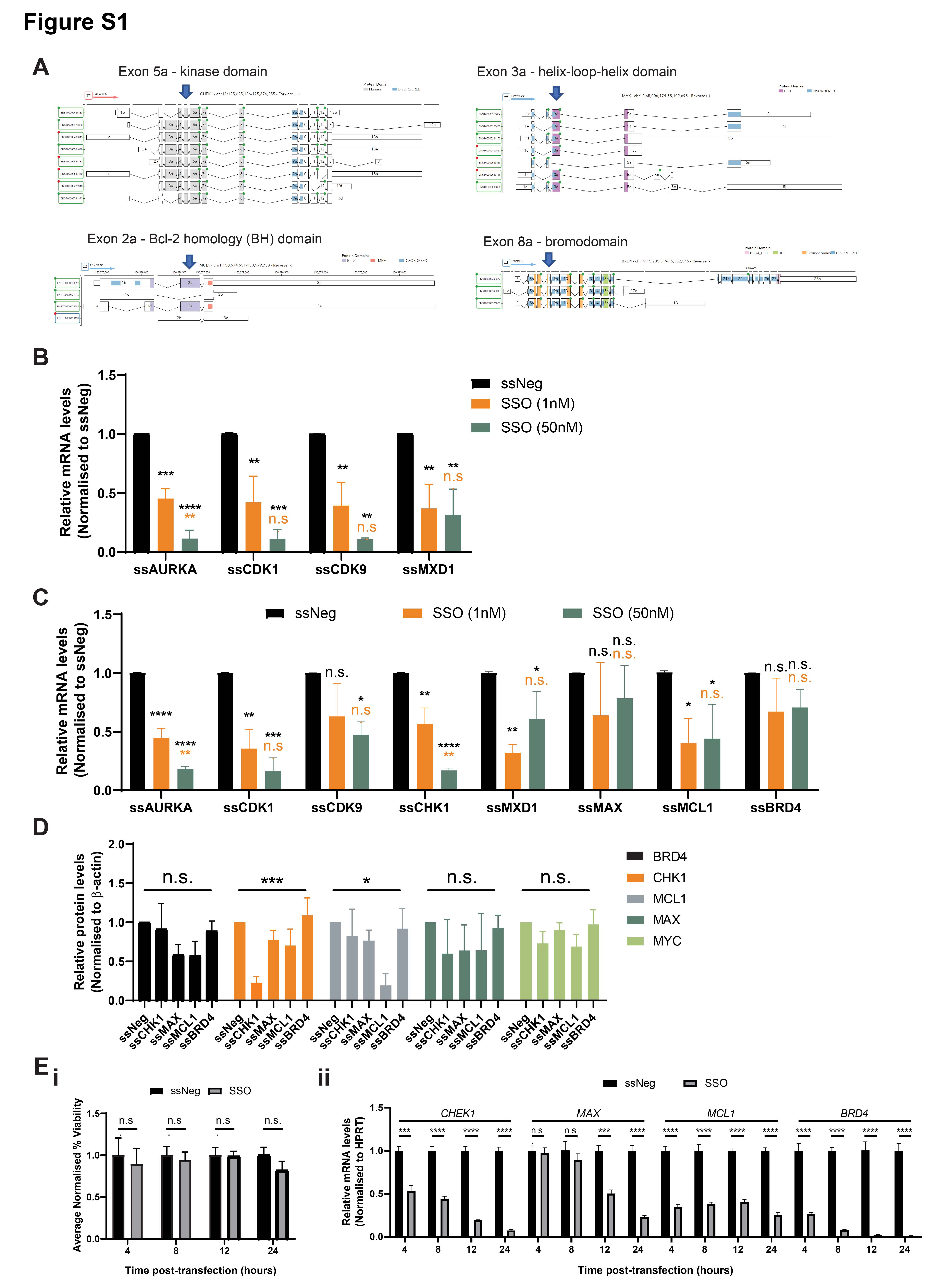

Supplement: Supplementary file 1 — Appendix S1 Supporting Information [file BTM2-8-e10363-s002.zip › Figure S1.jpg]

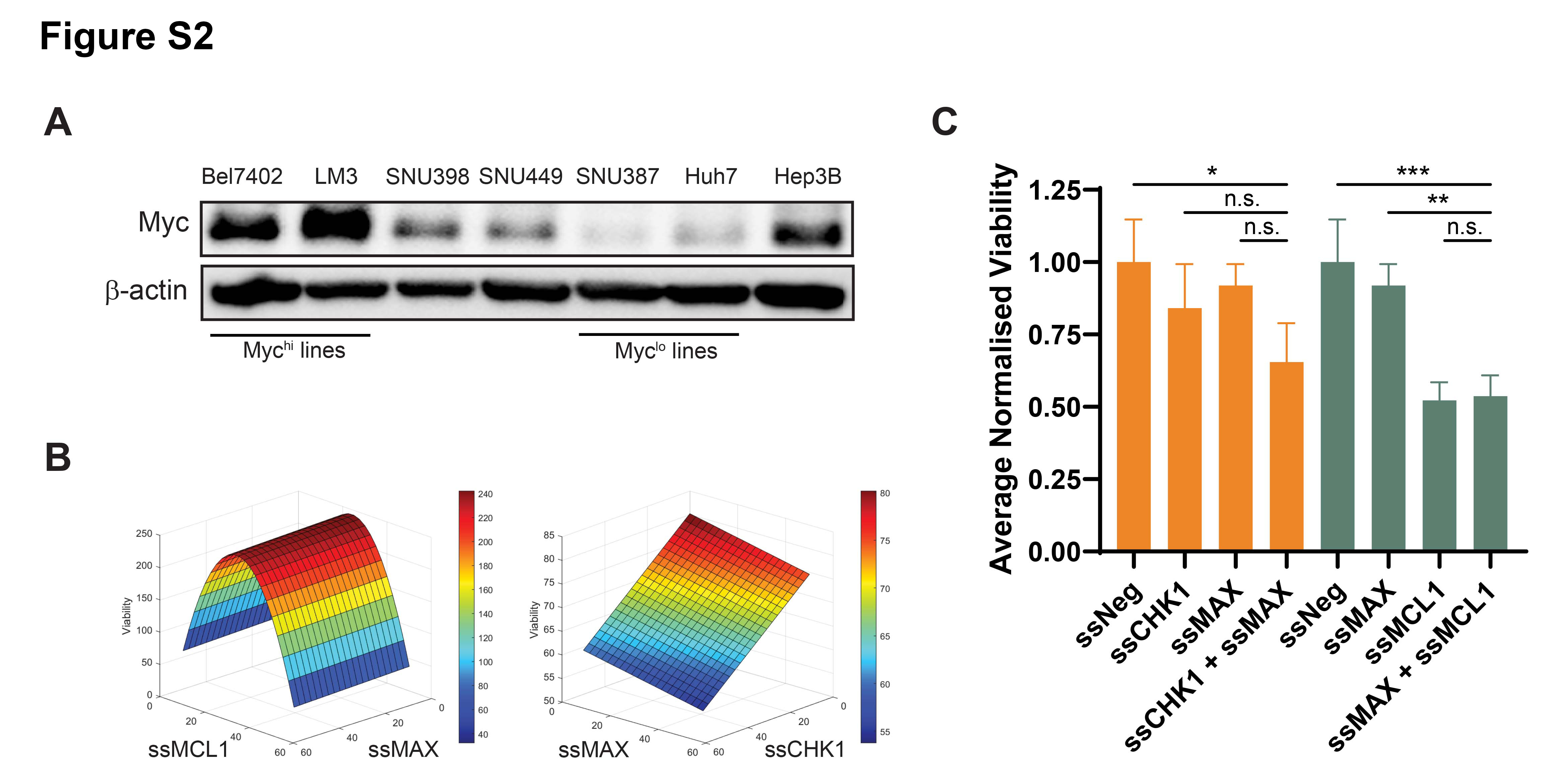

Supplement: Supplementary file 1 — Appendix S1 Supporting Information [file BTM2-8-e10363-s002.zip › Figure S2.jpg]

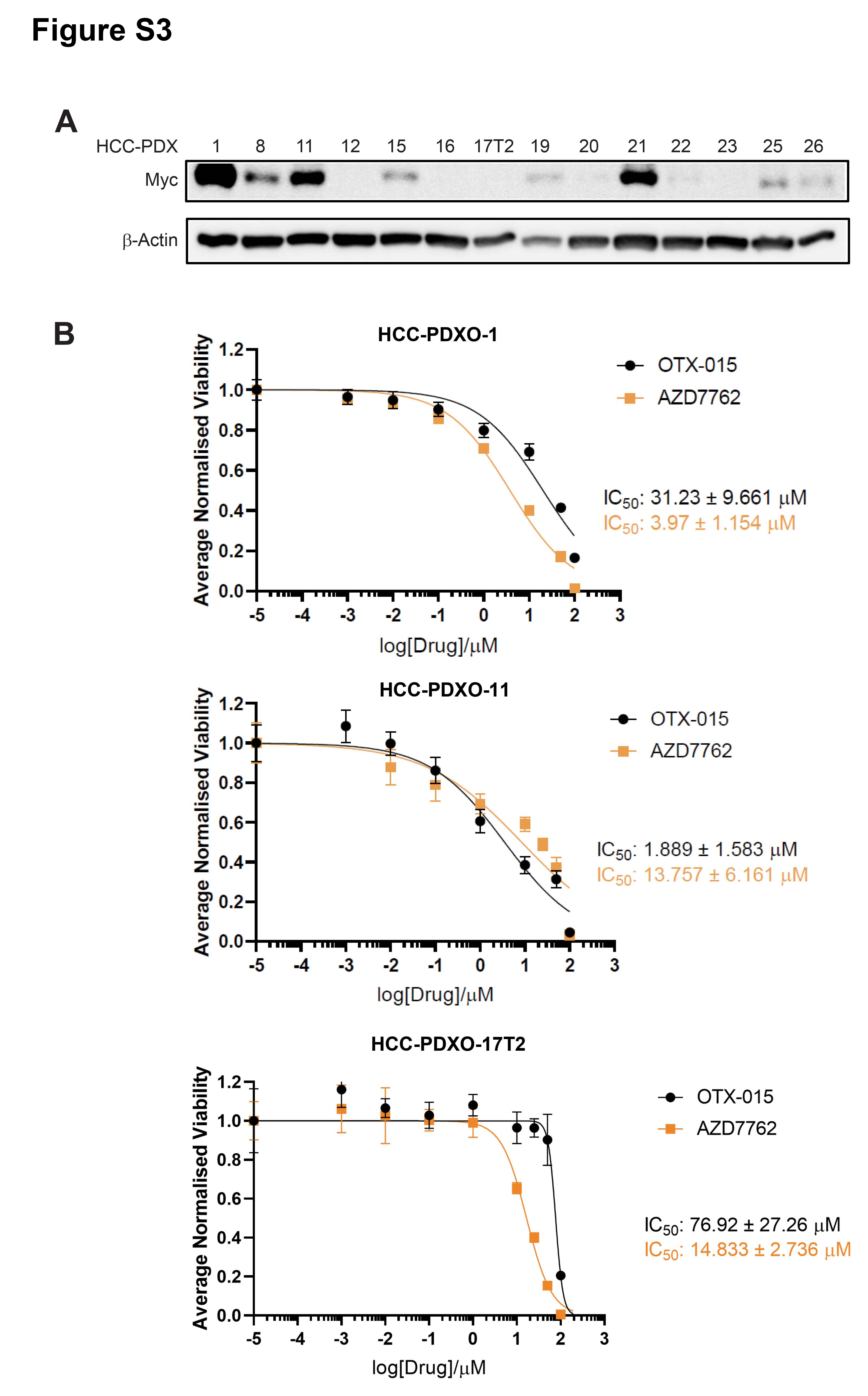

Supplement: Supplementary file 1 — Appendix S1 Supporting Information [file BTM2-8-e10363-s002.zip › Figure S3.jpg]

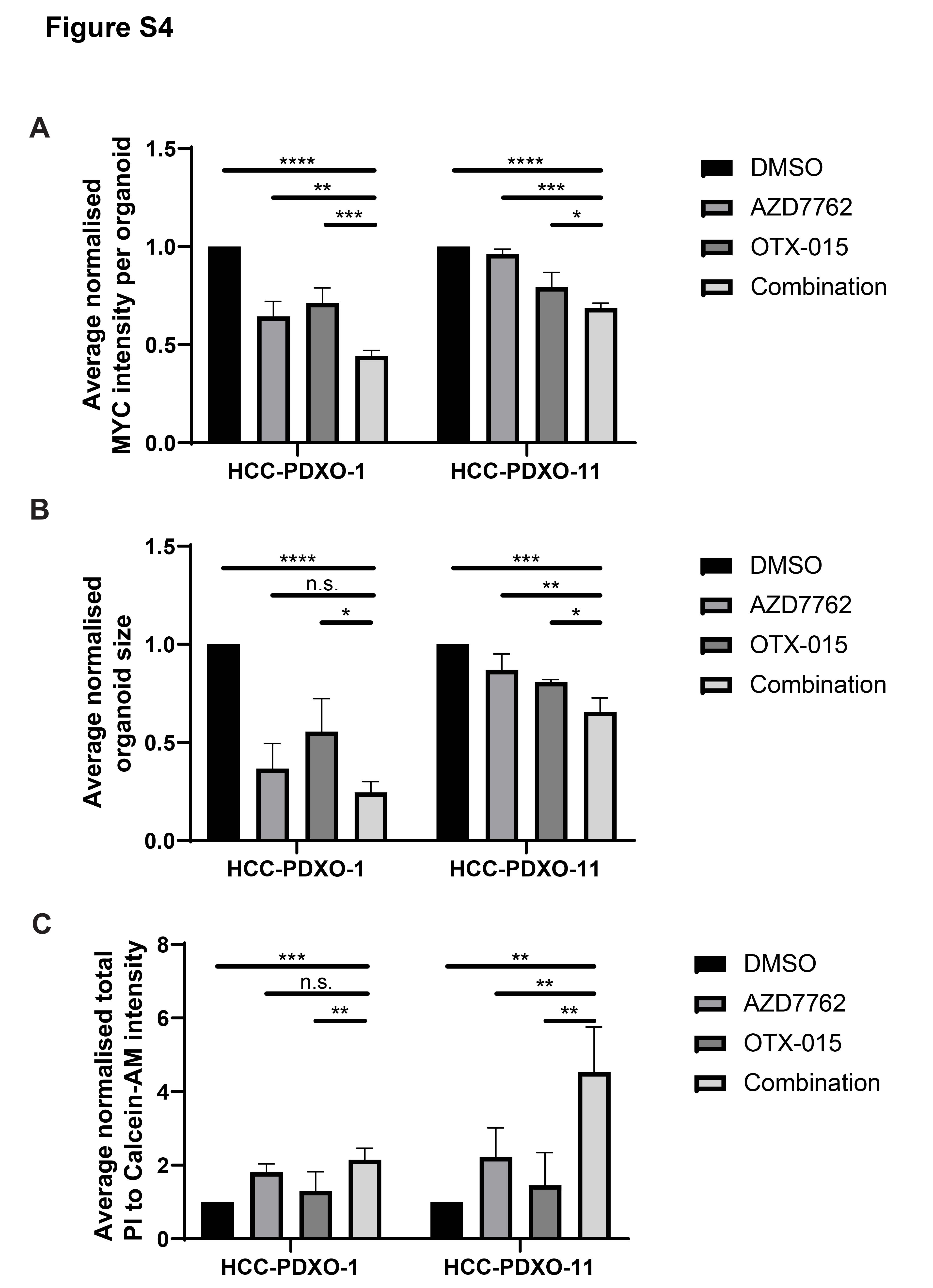

Supplement: Supplementary file 1 — Appendix S1 Supporting Information [file BTM2-8-e10363-s002.zip › Figure S4.jpg]
